# Arthroscopic Treatment of Chronic Cruciate Ligament Rupture in the Dog without Stifle Stabilization: 13 Cases (2001-2020)

**DOI:** 10.1155/2023/6811238

**Published:** 2023-04-11

**Authors:** Danielle G. Creamer, Peter Muir

**Affiliations:** Comparative Orthopaedic Research Laboratory, School of Veterinary Medicine, University of Wisconsin-Madison, Madison, WI 53706, USA

## Abstract

**Objective:**

The objective was to study clinical outcomes in dogs with chronic cruciate ligament rupture (CR) treated with palliative arthroscopy as the sole surgical treatment.

**Methods:**

Thirteen client-owned dogs with CR underwent physical examination, stifle radiography, and arthroscopy with resection of damaged meniscal tissue. Records were evaluated, and orthopaedic examination, radiographs, and arthroscopy images were assessed. Long-term clinical outcome was also assessed by use of an owner questionnaire.

**Results:**

Thirteen dogs that underwent arthroscopy at the UW Veterinary Care between 2001 and 2020 were included. Long-term follow-up was available for 7 of 13 dogs. Lameness was static to improved in all dogs in which arthroscopy was performed. Subsequent stifle stabilization was performed after arthroscopy in only 1 of 7 dogs with follow-up data.

**Conclusion:**

Palliative arthroscopy and resection of damaged meniscal tissue in combination with medical management of osteoarthritis can be considered in dogs with chronic CR and cranial tibial subluxation with little passive laxity during examination. Revision surgery with TPLO is uncommon after arthroscopy based on this study.

## 1. Introduction

Cruciate ligament rupture (CR) is one of the most common orthopaedic conditions and an important cause of chronic pelvic limb lameness in dogs. CR is a complex polygenic disease with genetic and environmental risk and moderate heritability [1, 2]. Prevalence of CR increases with age [3]. Larger dogs (>22 kg) are at greater risk of developing CR at a younger age [4]. In most dogs, CR has an insidious onset. As fiber rupture and increasing laxity develop in the cranial cruciate ligament (CrCL), degenerative changes develop within the stifle joint, including synovitis, osteoarthritis (OA), and periarticular fibrosis [5]. Fiber rupture in both the CrCL and the caudal cruciate ligament (CaCL) is typical [6]. Even with surgical intervention, such as tibial plateau leveling osteotomy (TPLO), progression of OA occurs [7]. However, TPLO remains the surgical stabilization with the best evidence of clinical efficacy [8].

Dogs with CR may be presented with lameness at differing clinical stages ranging from lameness in dogs with partial CR and a stable stifle with no palpable passive laxity to dogs with palpable passive laxity and periarticular changes and then to dogs with chronic CR and advanced periarticular changes such that the presence of palpable laxity is much reduced. Clinical diagnosis of CR early in the course of the disease is often missed because clinical signs may be subtle, and signs of passive laxity are absent or minimal. The presence of palpable stifle effusion [9], pain on stifle hyperextension or internal rotation, and periarticular fibrosis, particularly medial buttress, are important clinical signs of CR [10]. Radiographic stifle effusion and OA are also important diagnostic signs [9].

CR can be managed conservatively or surgically. Conservative management consists of strict activity restriction, physical therapy, weight loss, nutraceutical food supplements, and analgesic medication as indicated. For dogs ≤ 15 kg, conservative management can be successful resulting in a clinically normal gait with improvement in lameness [11]. For overweight dogs with CR, a combination of surgical and medical management has been found to have a higher probability of successful outcome compared to medical management alone [12]. Currently, surgical stabilization is the clinical treatment of choice for most dogs with CR. However, the role of stabilizing surgical treatment for dogs with established OA and periarticular fibrosis is unclear as clinical outcomes for this type of patient are poorly documented.

When long-term follow-up of dogs with CR treated with a lateral fabellar suture was performed, passive laxity in the group of dogs that were doing well clinically was found to be higher compared with the dogs that were doing poorly [13]. This suggests that surgical stabilization may be a less important determinant of clinical outcome in dogs with established OA and periarticular fibrosis after CR. Conversely, development of OA may be minimized when stabilizing treatment, such as TPLO, is performed early in the course of the disease [14].

The aim of the present study was to evaluate clinical outcomes in a case series of dogs presented with chronic CR with established OA and periarticular fibrosis treated with arthroscopy as the sole surgical treatment. Arthroscopic stifle examination is the current gold standard for the identification of cruciate ligament fiber damage, assessment of meniscal injury, and assessment of articular cartilage health, as it provides a direct magnified view of the surface of the ligaments and the menisci and enables probing of articular structures to assess tissue damage [15].

## 2. Materials and Methods

### 2.1. Dogs

**Figure 1:**
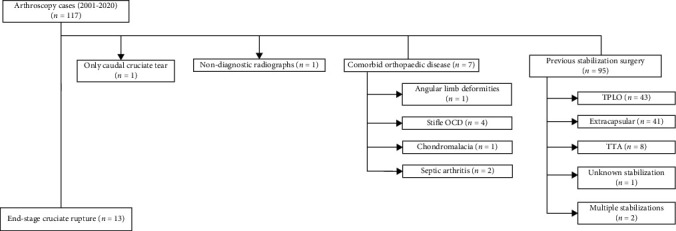
Flow diagram for case inclusion and exclusion. Of the 117 cases identified from the initial medical record search of arthroscopy cases, 13 dogs were included in the analysis.

Client-owned dogs with a diagnosis of CR that underwent arthroscopy were evaluated retrospectively between May 2001 to December 2020 at the University of Wisconsin-Madison UW Veterinary Care Hospital. Medical records of these dogs were screened to identify the subset of dogs that met study inclusion criteria (Figure 1). Dogs were included if they underwent stifle arthroscopy as part of their treatment but did not receive surgical stabilization of the stifle and had established stifle OA secondary to chronic CR. Dogs with a diagnosis of CR were excluded if previous stifle stabilization had been performed, if there was other stifle disease identified, and if diagnostic radiographs were not available in the medical record.

### 2.2. Clinical Examination

Physical examination findings were obtained from medical record data. Data collected included signalment, the owner's complaint, body weight (kg), current drug treatment, and lameness duration. If recorded in the medical record, the following data were obtained. The degree of lameness was scored from 0 (no detectable lameness) to 5 (nonweight bearing lameness during standing or walking) in the index pelvic limb [16]. Body condition score (0-9) was recorded. In addition, loss of stifle flexion or extension, the presence of stifle pain, crepitation, and muscle atrophy during examination, the presence of stifle effusion, and the severity of medial buttress or periarticular fibrosis (absent, 0; mild, 1; moderate, 2; severe, 3) were assessed [17]. Severity of cranial drawer and tibial thrust passive laxity was also assessed and graded (absent, 0; mild, 1; moderate, 2; and severe, 3) [17].

### 2.3. Radiographic Examination

Caudocranial and mediolateral radiographs of the clinical index stifle were reviewed as well as the contralateral stifle in dogs with bilateral CR. Severity of stifle joint effusion (0-2) and osteophytosis (0-3) were graded [9]. Cranial tibial subluxation was estimated from the mediolateral radiographic view by measuring the distance in millimeters between the caudal margin of the femoral condyles and the caudal cortex of the fibula [17]. Magnification was accounted for with a standard 10 cm measurement bar.

### 2.4. Arthroscopy

Arthroscopic images were reviewed, and grading of synovial hypertrophy, vascularity, and synovitis (0-4) was performed [15]. Fiber damage in the CrCL and the CaCL was also recorded (normal = 0, mild = 1, moderate = 2, severe = 3) after review of the patient's surgical report [15]. The presence of lateral and medial meniscus damage was recorded. Damage was assessed as present or absent, and the type of tear (bucket handle, radial, horizontal, peripheral detachment, and complex) was noted, if present.

### 2.5. Subjective Outcome Measures

A questionnaire was developed to evaluate long-term success of arthroscopy as the sole surgical therapy for chronic CR (Supplementary File S1). Owners of dogs included in the study were contacted to obtain long-term follow-up through completion of the questionnaire.

### 2.6. Statistical Analysis

Summary clinical data were reported including mean ± standard deviation.

## 3. Results

### 3.1. Clinical Examination

Dogs undergoing stifle arthroscopy were evaluated from 2001 to 2020. A total of 13 dogs were included after review of 117 cases (Figure 1). The population consisted of 10 females and 3 males; one male dog and one female dog were intact at the time of arthroscopy. The age was 5.3 ± 3.2 years (range 1-10 years, median 4 years). The body condition score was 6.6 ± 1.4 (range 4-8/9, median 7/9, *n* = 11). Body weight was 40.4 ± 11.8 kg (range 26.6-64 kg, median 37.3 kg). Breeds studied were mixed breed (2), Labrador Retriever (2), German Shepherd Dog (2), English Springer Spaniel (1), Newfoundland (2), Pit Bull Terrier (1), Golden Retriever (1), Rottweiler (1), and Bernese Mountain Dog (1).

Duration of lameness was variable and ranged from 1 month to 2 years. Mean duration of lameness was 0.9 ± 0.7 years (median 0.83 years). Current medical therapy at the time of arthroscopy included carprofen (4), carprofen and gabapentin (2), or firocoxib (1). Six of the dogs included in this study were not receiving medical therapy at presentation.

Severity of lameness was variable (Table 1). Lameness grade in the affected index limb was 2.1 ± 0.9 (*n* = 10, median 2, range 1-4). Lameness severity was not recorded in 3 dogs. Severity of joint pain and effusion was also variable. Moderate-to-severe medial buttress fibrosis was present in most dogs (Table 1). Loss of stifle flexion or extension was found in the 4 dogs in which this was reported, and a meniscal click was palpated in the five dogs in which this clinical sign was reported. Stifle crepitation was found in 4 of 5 dogs. Muscle atrophy was present in 6 of 8 dogs. Four dogs had absent cranial tibial thrust on physical examination, and two dogs had absent cranial drawer (Table 2). Severity of cranial drawer and tibial thrust were assessed as1.2 ± 0.7 (*n* = 13, median 1, range 0-2) and 1.1 ± 0.9 (*n* = 12, median 1, range 0-2), respectively (Table 2).

### 3.2. Radiographic Examination

**Figure 2:**
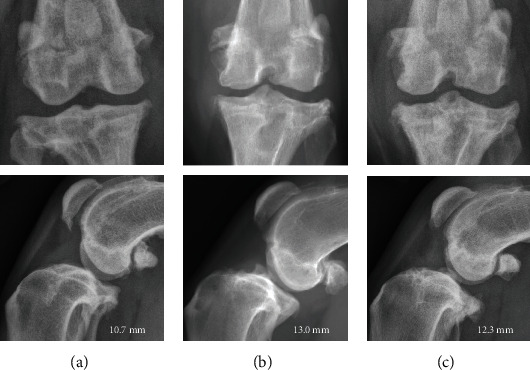
Representative orthogonal radiographic views of stifle from a 9-year-old female spayed Boxer mix (a), a 10-year-old female spayed Collie mix (b), and a 9-year-old male neutered Labrador Retriever (c) with severe osteoarthritis and synovial effusion in which arthroscopy was used as management for CR. Severity of stifle osteoarthritis and synovial effusion were graded based on previously published scales [9]. (a) and (b) represent grade 1 synovial effusion and grade 3 osteophytosis; (c) represents grade 2 synovial effusion and grade 3 osteophytosis. In images (b) and (c), the medial buttress is moderate to severe. The mm of tibial translation is noted in each image.

Synovial fluid effusion and OA ranged between 1-2 and 1-3, respectively (Table 2 and Figure 2). Cranial tibial subluxation was assessed on mediolateral radiographs. Cranial tibial subluxation ranged from 5.8 mm to 18.5 mm. Mean cranial tibial subluxation was 11.7 ± 3.0 mm (median 12.4 mm). Two dogs did not have cranial drawer laxity identified on physical examination, but had 13.2 mm, and 13.5 mm of radiographic cranial tibial translation, respectively, while 11 dogs had positive cranial drawer with a range of 5.8-18.5 mm of cranial tibial translation radiographically (Table 2).

### 3.3. Arthroscopy

#### 3.3.1. Synovial Changes

**Figure 3:**
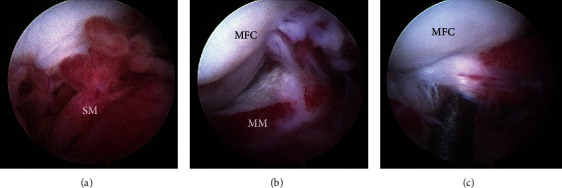
Arthroscopic images from a 6-year-old male intact German Shepherd Dog with severe osteoarthritis and synovial effusion in which arthroscopy was used as management for CR. Synovitis was graded based on previously published guidelines [15]. These images represent severe hypertrophy, vascularity, and synovitis. (a) Inflamed synovial membrane with enlarged synovial villae, (b) arthroscopic view of the medial aspect of the intercondylar notch, and (c) medial femoro-tibial joint with a chronic meniscal tear. Note: SM: synovial membrane; MFC: medial femoral condyle; MM: medial meniscus.

Severity of synovitis and vascularity ranged from 1 to 4, while hypertrophy ranged from 2 to 4 (Figure 3 and Table 3). Synovitis and vascularity grade was 2.9 ± 0.9 (median 3), and hypertrophy grade was 3.3 ± 0.8 (median 3). One dog had grades of 1 for both vascularity and synovitis while all other dogs had grades greater than 1 for all aspects of arthroscopic grading of synovial changes. Two of the dogs did not have arthroscopic images to review.

#### 3.3.2. Ligament Fiber Damage

Median CrCL fiber damage was 3 and ranged from 0 to 3 in the 11 dogs with arthroscopic data. In one dog, CrCL tissue was absent suggesting complete ligament resorption. No tibial thrust and mild cranial drawer were found on examination of this patient. Median CaCL fiber damage was 1 and ranged from 0 to 3. CaCL fiber damage was not identified in 5 dogs.

#### 3.3.3. Meniscus

Caudal pole bucket handle tearing of the medial meniscus was found in 8 dogs. Complex medial meniscal tears were found in 2 dogs. The lateral and medial menisci were completely resorbed in one dog. The lateral and medial menisci were both intact in one dog. Tearing of the cranial pole of the lateral meniscus was found on one dog that had previously had a medial meniscectomy performed. Resection of the damaged tissue was performed in all dogs found to have meniscal injury.

### 3.4. Owner Follow-Up and Questionnaire

Owners of the 13 dogs were contacted, and 6 dogs were lost to follow-up. Full questionnaire follow-up was provided for 4 of the 7 dogs. Incomplete verbal discussion was obtained for 3 dogs. No further stifle surgery was performed on 6 of the 7 dogs, of which three had absent tibial thrust. Subsequent surgical stabilization was performed at the UW Veterinary Care for 1 of the 7 dogs. The one dog which had additional surgery was a Bernese Mountain Dog with a duration of lameness of 5 months. There was moderate cranial drawer and tibial thrust as well as a grade 2/5 lameness and moderate loss of range of motion and pain on physical examination. Radiographic evaluation of the stifle revealed mild synovial effusion and osteophytosis. Radiographic cranial tibial subluxation was 5.8 mm. Arthroscopy of the stifle confirmed cranial cruciate ligament damage and no meniscal damage. Synovial assessment identified moderate hypertrophy, mild vascularity, and mild synovitis. After the initial arthroscopic surgery, lameness improved based on the questionnaire responses and was mild one year after surgery. Ultimately, TPLO treatment was pursued two years after the initial arthroscopic treatment.

After arthroscopy, 5 dogs had clinical improvement, including 2 dogs with absent tibial thrust. Four of these dogs had mild to no lameness beyond one year after arthroscopy. The other dog that was reported to be clinically improved had a moderate lameness > 1 year after arthroscopy. This dog, a 1-year-old female spayed Newfoundland, with absent cranial drawer and tibial thrust on physical examination, had moderate lameness after arthroscopy and was available for long-term follow-up. The dog initially presented with bilateral CR and bilateral grade 3/5 lameness. Cranial drawer and tibial thrust were present in both pelvic limbs. The more severely affected limb clinically had a TPLO performed. Seven months later, the dog was reexamined, and arthroscopy was performed on the contralateral stifle. At this time, there was absent cranial drawer and tibial thrust with marked periarticular fibrosis of the stifle. This dog had radiographic tibial translation of 13 mm. After both arthroscopy of this stifle and TPLO treatment of the contralateral stifle, lameness was described as moderate.

One dog was considered to have lameness of similar severity after arthroscopy. The dog, which was perceived to be static by the owner, originally presented with mild lameness that remained mild after arthroscopy. This dog was a 9-year-old female spayed Border Collie cross that was presented with bilateral CR and a two-year history of bilateral pelvic limb lameness. Upon physical examination, the dog had negative tibial thrust bilaterally. The study limb had radiographic tibial translation of 13.0 mm. Bilateral stifle arthroscopy was performed, and no further stifle stabilization was pursued at long-term follow-up.

Treatment with pain-relieving medication was used in 5 of 7 dogs, including the Border Collie cross that did not improve clinically with arthroscopic treatment. Daily carprofen was used in the Newfoundland with moderate lameness after arthroscopy as well as the Border Collie cross. Other medications used were intermittent carprofen, gabapentin, or tramadol beyond one year after arthroscopy.

Three of the 7 dogs had bilateral CR at the time of arthroscopic surgery. Two of these dogs had developed CR of the contralateral limb before arthroscopy, and earlier stifle stabilization using TPLO was performed. Two dogs had bilateral CR at the time of initial presentation. One had bilateral stifle arthroscopy performed, while the other had stifle stabilization using a TPLO on the most clinically severe limb. Arthroscopy of the contralateral limb was performed 1 year later. In the third dog with bilateral CR that received arthroscopic treatment, development of contralateral CR occurred according to the owner questionnaire at one year after arthroscopy. Per UW Veterinary Care records, TPLO was used as treatment for the contralateral CR.

## 4. Discussion

The present study evaluated arthroscopy as the sole salvage treatment for palliative management of joint pain in 13 dogs with chronic CR. Most were neutered large breed adult dogs, consistent with published risk data [4, 18]. Duration of lameness at presentation was variable and was not associated with severity of synovitis and OA. Synovitis is a key feature of partial CR stifles before development of stifle laxity that is correlated with radiographic OA [5]. A bucket handle tear or complex damage of the medial meniscus was commonly found at surgery, consistent with other published data [19]. At the time of arthroscopy, ~50% of the dogs were receiving pain-relieving medication, particularly nonsteroidal anti-inflammatory medication, to address chronic lameness because residual lameness is common after stifle stabilization surgery [20].

Cranial drawer and tibial thrust were noted on physical examination, and cranial tibial translation was estimated from radiographs. Several of the dogs had little or no palpable laxity on physical examination, but obvious radiographic cranial tibial subluxation. This common scenario reflects the fact that progressive periarticular fibrosis over time may lead to fixation of the tibia in a displaced position. One study found that long-term chronic pain was present in 30% of dogs after surgical treatment for CR after a mean of 2.7 years using a validated Helsinki chronic pain index (HCPI) [21]. This was corroborated in another study in which one third of dogs evaluated 1-5 years after surgical repair had a pain response on flexion and/or extension of the stifle, and 20% had a lameness of the affected limb [22]. Similar to earlier observations [13] kinematic analysis of the stifle after lateral fabellar suture treatment of cruciate rupture showed clinical improvement in lameness, but persistent cranial tibial subluxation [23].

These studies suggest that synovitis and OA are the major source of joint pain in dogs with chronic CR, rather than passive laxity associated with persistent cranial tibial subluxation or dynamic instability during weight-bearing. Additionally, the potential cause-effect relationship between stifle synovitis and progressive fiber rupture in the cruciate ligament complex is not fully understood [5]. What constitutes optimal anti-inflammatory and analgesic medication for this type of patient remains to be determined.

Of the 7 of 13 dogs for which follow-up was available, only one dog underwent subsequent stifle stabilization. This dog had the smallest amount of radiographic tibial subluxation for the group of dogs in this report and was the only one with intact menisci.

In our study, four dogs had absent cranial tibial thrust, and two other dogs had absent cranial drawer. Radiographic tibial translation in these dogs ranged from 13 to 14 mm. This suggests that periarticular fibrosis had largely resolved passive stifle laxity with the joint becoming fixed in a subluxated position. Based on our study, we hypothesize that chronic CR dogs with cranial tibial subluxation and periarticular fibrosis are poor candidates for stifle stabilization treatment and even with stabilization may continue to have chronic stifle pain. More work is needed to evaluate long-term outcomes of stabilization procedures in such cases. Rather than stifle stabilization, treatment with joint replacement surgery is potentially indicated in dogs with chronic CR and advanced OA and lameness that is unresponsive to meniscectomy or medical treatment.

There are several limitations associated with this work. This retrospective study reviewed case material over a nineteen-year period. Owners of 6 of the 13 dogs were not contactable and were lost to follow-up. The relatively small sample size and incomplete long-term follow-up limits our conclusions. Some missing medical record data also influenced the quality of the data set.

In conclusion, chronic CR is often associated with development of relatively fixed cranial tibial subluxation because of periarticular and capsular fibrosis. Such dogs may have little to no palpable passive laxity during stifle clinical examination, and meniscal damage is typical. Such patients represent a treatment challenge. Palliative arthroscopic evaluation of the stifle and resection of damaged meniscal tissue combined with medical management of OA offers a reasonable prognosis, and revision surgery with TPLO is uncommon after this type of treatment based on our study and long-term follow-up of 7 dogs. Long-term treatment with oral medical treatment may be needed to manage lameness.

## Figures and Tables

**Table 1:** Clinical examination findings in 13 dogs treated with arthroscopy for chronic CR.

Patient	Signalment	BCS	Medial buttress	Loss of range of motion	Lameness of the affected limb	Stifle effusion	Joint pain	Meniscal click	Stifle crepitation	Muscle atrophy
1	10 yo FS Collie mix	7/9		1			0		1	0
2	9 yo FS Boxer mix	5/9	2	2	4/5	0	2	1	0	1
3	6 yo MI German Shepherd Dog	4/9	2-3		2/5	1	2			0
4	4 yo FS German Shepherd Dog		2		2/5	2				1
5	2 yo FS Newfoundland	8/9	3		1/5	3	2	1	1	1
6	8 yo FS English Bulldog	6/9	2		1/5	0		1		0
7	9 yo MN Labrador Retriever	8/9	3		3/5^∗^	1	2			1
8	3 yo FS Rottweiler	5/9			2/5				1	
9	1 yo FS Newfoundland	8/9	2	2		2			1	
10	4 yo FS Labrador Retriever		2		3/5	2	2			
11	2 yo FS Pit Bull Terrier mix	7/9			2/5			1		1
12	2 yo FI Bernese Mountain Dog	6/9	1	2	2/5	1	2			1
13	9 yo MN Golden Retriever	8/9	2			2	2	1		

Note. Body condition score out of 9 was recorded as well as the presence of medial buttress, range of motion of the stifle joint, lameness, stifle effusion, and pain. Lameness grade at presentation was recorded (0 (no detectable lameness) to 5 (nonweight bearing lameness during standing or walking)) [16]. Pain, medial buttress, and loss of range of motion were either not stated or graded (absent = 0, mild = 1, moderate = 2, severe = 3) [17]. Meniscal clicking, stifle crepitation, and muscle atrophy were recorded as present (1) or absent (0). Empty cells in the table represent missing data. ^∗^Lameness in this dog included lateral excursion.

**Table 2:** Severity of palpable stifle passive laxity, radiographic cranial tibial subluxation, and radiographic osteoarthritis in 13 dogs with chronic cruciate ligament rupture.

Patient	Cranial tibial thrust	Cranial drawer	Procedure	Severity of synovial effusion	Severity of osteophytosis	Radiographic cranial tibial subluxation (mm)
1	0	2	Bilateral stifle arthroscopy	1	3	13.0
2	1	1	Right stifle arthroscopy and arthrotomy with partial meniscectomy	1	3	10.7
3	0	1	Left stifle arthroscopy	2	2	12.7
4	0	1	Right stifle arthroscopy and meniscectomy	1	3	13.4
5	1	1	Bilateral stifle arthroscopy and meniscectomy	2	2	10.4
6	2	2	Bilateral arthroscopy and meniscectomy with hyaluronic acid injections	1	3	18.5
7	2	2	Bilateral stifle arthroscopy and meniscectomy with hyaluronic acid injections	2	3	12.3
8	2	1	Right stifle arthroscopy and meniscectomy	1	3	7.3
9	0	0	Right stifle arthroscopy and hyaluronic acid injection	2	3	13.2
10	2	2	Right stifle arthroscopy and arthrotomy with meniscectomy	2	3	12.4
11		0	Left stifle arthroscopy and arthrotomy with meniscectomy	2	3	13.5
12	2	2	Right stifle arthroscopy with PRP injection	1	1	5.8
13	1	1	Right stifle arthroscopy and arthrotomy	2	3	9.4

Note. Cranial tibial subluxation was estimated using an established method [17]. Severity of passive laxity was graded as absent (0), mild (1), moderate (2), and severe (3). Empty cells in the table represent missing data.

**Table 3:** Arthroscopic grading of synovial pathology and cruciate ligament fiber rupture in dogs with chronic cruciate ligament rupture.

Patient	Hypertrophy	Vascularity	Synovitis	CrCL damage	CaCL damage
1	3	2	3	3	0
2	4	3	4	3	0
3	4	4	4	3	3
4	3	3	3	3	3
5	4	3	4	3	0
6	4	3	2	3	2
7	2	2	2	2	0
8	3	4	3	3	1
9	3	3	3	2	0
10	4	4	4	3	2
12	2	1	1	1	

Note. Arthroscopic images from 11 of 13 patients were reviewed based on previously published guidelines [15]. Arthroscopic images were not available for dogs 11 and 13. Fiber damage in the cranial cruciate ligament (CrCL) and the caudal cruciate ligament (CaCL) was recorded (normal = 0, mild = 1, moderate = 2, severe = 3) after review of the patient's surgical report and arthroscopic images [17]. Empty cells in the table represent missing data.

## Data Availability

The clinical data used to support the findings of this study are included within the article.

## Supplementary Materials

Supplementary Materials Supplementary File S1. Owner questionnaire for dogs with a history of chronic cranial cruciate ligament rupture, treated with partial or complete meniscectomy as appropriate.
