# Supplementary material for: Arthroscopic Treatment of Chronic Cruciate Ligament Rupture in the Dog without Stifle Stabilization: 13 Cases (2001-2020)
Source: Case Rep Vet Med. 2023 Apr 11;2023:6811238. doi: 10.1155/2023/6811238 (PMC10113050; doi:10.1155/2023/6811238)
Supplement: Supplementary Materials — Supplementary File S1. Owner questionnaire for dogs with a history of chronic cranial cruciate ligament rupture, treated with partial or complete meniscectomy as appropriate. [file 6811238.f1.zip › Owner Survey Chronic CR.docx]

**Comparative Genetics and Orthopaedic Research Laboratory**

**School of Veterinary Medicine**

**
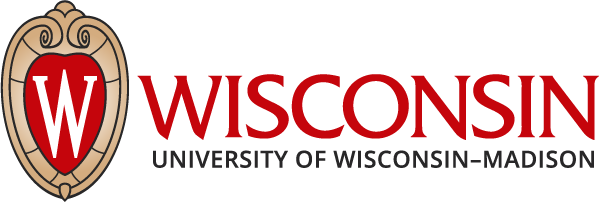
**

**Owner questionnaire for dogs with a history of chronic cranial cruciate ligament rupture, treated with meniscal removal**

Dear Owner,

Thank you for agreeing to complete this questionnaire.

Your assistance in this endeavor will enable us to gather valuable information about your pet, and is a vital component in our ongoing quest to understand the optimal way to treat animals with chronic cranial cruciate ligament rupture. It is important that all questions are answered to the best of your ability. If you have a question regarding the questionnaire, please ask.

Answering the questions

Most of the questions are fairly simple. It is important that you should **only tick one box per question**, except where otherwise requested.

To be filled out by staff:



**I. Patient Information**
Your Name: ___________________ Today’s Date: ____________

Your Dog’s Name: __________________ Date of Birth: _______________

Breed: ____________________ Color: __________________

Weight: ___________________ (lb/kg – circle one)

Sex:(please check one answer)

  Female  Spayed Female  Male  Neutered Male

If your dog is spayed/neutered - Is the date of the spay/neuter known?

 Yes - If yes, please list date (month/day/year) : ______________________

 No - If no, is approximate age range (at the time of spay/neuter) known?

 Yes - If yes, please list approximate age: _________________

 Unknown

Has your dog been diagnosed with any other orthopedic problems?

 No  Yes If yes, please list if you can with date of diagnosis

1 . . . . . . . . . . . . . . . . . . . . . . . . . . . . . . . . . . . . . . . . . . . . . . . . . . . . . . . . . . . . . .

2 . . . . . . . . . . . . . . . . . . . . . . . . . . . . . . . . . . . . . . . . . . . . . . . . . . . . . . . . . . . . . .

3 . . . . . . . . . . . . . . . . . . . . . . . . . . . . . . . . . . . . . . . . . . . . . . . . . . . . . . . . . . . . . .

Has your dog been diagnosed with Cushing’s disease (hyperadrenocorticism)?

 No  Yes (if so, when:________________)

Has your dog ever been on long-term systemic steroid therapy (e.g. prednisone for treatment of

immune-mediated disease)?

 No  Yes (if so, when:_______________)

Has your dog been diagnosed with any other non-orthopedic problems (e.g. cancer)?

Please list if you can with date of diagnosis

1 . . . . . . . . . . . . . . . . . . . . . . . . . . . . . . . . . . . . . . . . . . . . . . . . . . . . . . . . . . . . . .

2 . . . . . . . . . . . . . . . . . . . . . . . . . . . . . . . . . . . . . . . . . . . . . . . . . . . . . . . . . . . . . .

3 . . . . . . . . . . . . . . . . . . . . . . . . . . . . . . . . . . . . . . . . . . . . . . . . . . . . . . . . . . . . . .

**II. History for the Stifle/Knee with chronic cruciate ligament rupture, treated with meniscus removal**

Our records indicate that your dog had a chronic cruciate rupture that was best treated by surgical debridement of the meniscus without a stabilization procedure at UW Veterinary Care. Please answer the following questions to the best of your ability. If you have other comments or clarifications, there is room to write more at the end of this questionnaire.

1. Date or age at the time of CrCL diagnosis in limb that only had meniscus removal: ______________

*This is the date of initial diagnosis, not the date of surgical intervention

2. Lameness of the operated limb immediately prior to surgical procedure: *(please select one)*

 Mild (mild limp, still able to walk well)

 Moderate (obvious limp, but still willing to put some weight on leg)

 Severe (toe-touching or not willing to put weight on leg)

3. Lameness of the affected limb 2-3 months after surgery: *(please select one)*

 Mild (mild limp, still able to walk well)

 Moderate (obvious limp, but still willing to put some weight on leg)

 Severe (toe-touching or not willing to put weight on leg)

4. Lameness of the affected limb 1+ years after surgery: *(please select one)*

 Mild (mild limp, still able to walk well)

 Moderate (obvious limp, but still willing to put some weight on leg)

 Severe (toe-touching or not willing to put weight on leg)

5. Did your dog eventually have additional surgery to stabilize this knee?

 Yes  No

If YES:

5a. What type of stabilization surgery was undertaken?

 TPLO  TTA  Extracapsular (suture repair)  Don’t know

5b. When was this surgery undertaken? ____________________

5c. Did this stabilization surgery improve your dog’s lameness?

 Yes  No

6. Did your dog ever rupture his/her cruciate on the *other* leg?

 Yes  No

If YES:

6a. When was this rupture diagnosed? (please write date or age): ________________

6b. Did this knee receive surgical treatment or stabilization, if so what type?

 Yes  No

If YES:

6b1.  TPLO  TTA  Extracapsular (suture repair)  Don’t know

**III Pain Relieving Medications**

Your dog was likely sent home with pain-relieving medications after his/her surgery for 7-10 days. Many dogs continue use of pain-relieving medications over the long term. These questions relate to use of pain-relieving medications beyond 7-10 days after surgery.

7. Most dogs are sent home with a NSAID medication that is recommended to be used long term. What is the best description of your administration of non-steroidal anti-inflammatory drugs (NSAIDs) such as Rimadyl, Vetprofen, Carprofen, Meloxicam, Previcox, Desuquin or aspirin ***for the 1^st^ year*** after surgery?

 We used an NSAID medication at least 5 times a week

 We used an NSAID medication on an as-needed basis

Please list often you gave your dog an NSAID (on average): _____________ per week / month

 We tried to use an NSAID medication long term but our dog did not tolerate this medication well (see below for further questions about other medications used)

 We tried this medication but did not think it was helping our dog so we stopped (please note below how long you tried the NSAID medication)

 We were sent home with an NSAID but did not use an NSAID medication long term

 We were never sent home with an NSAID medication

7a: Please note which NSAID medication was used (if applicable): ______________________

8. Some dogs are sent home with pain-relieving medications other than, or in addition to, NSAIDs. Did you give any of the following prescription pain-relieving medications for the ***1^st^ year after*** surgery, including: acetaminophen with codeine (Tylenol 4), acetaminophen (Tylenol), tramadol, gabapentin or pregabalin?

 Yes, we used at least one of these medications after surgery at least 5 times a week

 Yes, we used at least one of these medications on an as-needed basis

Please list how often you gave your dog medication: ________________ per week / month

 Yes, we tried to use pain-relieving medication long term but our dog did not tolerate this medication well

 No, we did not use any of these medication long term

 No, we were never sent home with an one of these medications

8a. Please note which medications were used if applicable: ______________________

Please comment on whether NSAID or other medications were used, and how they were used, beyond 1 year after surgery (if applicable):

________________________________________________________________________________

________________________________________________________________________________

________________________________________________________________________________

Please use the room below to add any details or explanations of the above questions. Please also add any additional information that you feel would be helpful for us to know about your dog!

___________________________________________________________________________

___________________________________________________________________________

___________________________________________________________________________

___________________________________________________________________________

___________________________________________________________________________

___________________________________________________________________________

___________________________________________________________________________

___________________________________________________________________________

___________________________________________________________________________

**Thank you again for completing this questionnaire.**
