# Supplementary material for: Arthroscopic Treatment of Chronic Cruciate Ligament Rupture in the Dog without Stifle Stabilization: 13 Cases (2001-2020)
Source: Case Rep Vet Med. 2023 Apr 11;2023:6811238. doi: 10.1155/2023/6811238 (PMC10113050; doi:10.1155/2023/6811238)
Supplement: Supplementary Materials — Supplementary File S1. Owner questionnaire for dogs with a history of chronic cranial cruciate ligament rupture, treated with partial or complete meniscectomy as appropriate. [file 6811238.f1.zip › Supplementary Description.docx]

**Supplementary File**

**Supplementary File S1**. Owner questionnaire for dogs with a history of chronic cranial cruciate ligament rupture, treated with meniscal removal.
